# Distinct Role of Striatal Functional Connectivity and Dopaminergic Loss in Parkinson’s Symptoms

**DOI:** 10.3389/fnagi.2017.00151

**Published:** 2017-05-23

**Authors:** Juergen Dukart, Fabio Sambataro, Alessandro Bertolino

**Affiliations:** ^1^Roche Pharmaceutical Research and Early Development, Neuroscience, Ophthalmology and Rare Diseases, Discovery and Translational Area, Roche Innovation Center BaselBasel, Switzerland; ^2^Department of Experimental and Clinical Medical Sciences, University of UdineUdine, Italy; ^3^Department of Basic Medical Science, Neuroscience and Sense Organs, University of Bari Aldo MoroBari, Italy

**Keywords:** striatal functional connectivity, DAT-SPECT, Parkinson’s disease, clinical symptoms, Unified Parkinson’s Disease Rating Scale

## Abstract

Degeneration of dopaminergic neurons is a hallmark of Parkinson’s disease. However, its link to Parkinson’s disease symptoms remains unclear. Striatal resting state functional connectivity differentiates between Parkinson’s disease patients and healthy controls and might be a potential mediator of the effects of striatal dopaminergic degeneration onto Parkinson’s disease symptoms. Here, we evaluated the relationship between dopaminergic deficits, striatal functional connectivity (SFC) at rest and different Parkinson’s disease clinical symptoms in the largest currently established cohort of *de novo* Parkinson’s disease patients. We show that SFC is an independent predictor of symptom severity in Parkinson’s disease in addition to striatal dopaminergic deficits. Furthermore, we find that distinct SFC networks are associated with symptoms reflecting the ability to perform daily routine automatized motor tasks and clinician-rated Parkinson’s disease motor symptoms. We find that reduced SFC is a major and independent predictor of Parkinson’s disease symptoms going beyond the mere reflection of striatal dopaminergic input loss. These findings indicate the high value of SFC as a clinically relevant biomarker in Parkinson’s disease.

## Introduction

Parkinson’s disease (PD) is a progressive neurodegenerative disorder that is associated with loss of dopaminergic projections from substantia nigra to the striatum. This decline of dopamine signaling initiates a pathological cascade encompassing numerous brain regions (Lotharius and Brundin, 2002; Dauer and Przedborski, 2003). Though loss of dopaminergic neurons as measured for example by dopamine transporter single photon emission tomography (DAT-SPECT) is consistently found to be correlated with PD motor symptoms at advanced disease stages, data on its association to symptom severity in *de novo* PD is rather limited (Pirker, 2003; McGhee et al., 2013). Also the contribution of different pathology levels and brain anatomical networks to the observed motor and non-motor symptoms covered by the Unified Parkinson’s Disease Rating Scale (UPDRS) and its revised version (MDS-UPDRS) – the most established PD rating scale – remains rather unclear (Fahn and Elton, 1987; Ramaker et al., 2002; Goetz et al., 2008). Subscales of the original and revised UPDRS scale have been repeatedly shown to reflect differential clinical aspects observed in PD patients (Martinez-Martin et al., 1994; Stebbins and Goetz, 1998; Goetz et al., 2008). UPDRS part I comprises symptoms related to mood and physiology – functions that are rather associated with prefrontal, insular and brain stem circuitry (Murai et al., 2001; Wolters, 2009; Benoit and Robert, 2011). UPDRS part II covers self-evaluation of highly automatized motor aspects of daily living. Retention of such automatized motor functions is closely associated with a striato-cerebellar circuitry (Doyon et al., 1998; Lang and Bastian, 2002). In contrast, UPDRS part III provides a direct evaluation of current motor deficits by the clinician. For this subscale, a stronger involvement of striatal, motor, premotor and prefrontal regions involved in execution and planning is therefore expected (Goldberg, 1985; Doyon et al., 1998; Petrides, 2005). A common mechanism underlying all symptoms captured by UPDRS subscales appears therefore unlikely.

The current knowledge regarding these potentially differential mechanisms underlying PD symptoms is rather limited. Evidence from single photon and positron emission tomography studies indicates that striatal dopaminergic signal accounts for up to 25% of variance in UPDRS total and its motor related part III subscale in advanced PD [for a detailed review see (McGhee et al., 2013)]. In contrast, the evidence for a link to UPDRS part I and II remains sparse. More recently, resting state functional magnetic resonance imaging (rsMRI) studies in PD reported significant associations between UPDRS part III and different striatal functional connectivity (SFC) metrics, explaining a similar proportion of variance as found for dopaminergic tracers (Wu et al., 2011; Hacker et al., 2012). At the same time several studies found a correlation between striatal different striatal connectivity metrics and striatal DAT binding (Lebedev et al., 2014; Rieckmann et al., 2015). These findings suggest that SFC desynchronization could be a further more downstream key pathological mechanism contributing to the observed PD motor symptoms (Ham et al., 2015). However, these studies did not evaluate if SFC provides any additional contribution to PD symptoms when accounting for loss of dopaminergic neurons. Correspondingly, the observed correlations could be due to the dopaminergic signal loss inducing both reductions in SFC and increases in symptom severity without a direct contribution of SFC to PD symptoms. The generalizability of these findings is also rather limited due to small sample sizes, inclusion of mostly advanced PD patients and insufficient control for potential treatment and atrophy effects onto the extracted resting state measures and the unclear link to other PD symptom domains.

Here we aim to address the question of differential contribution of dopaminergic deficits and SFC to PD clinical symptoms in a *de novo* PD population. We evaluate associations between dopaminergic loss, SFC and clinical symptoms as measured by the revised UPDRS (further referred to as UPDRS). Based on the above arguments, we hypothesize that reductions in SFC contribute to PD symptoms beyond the mere loss of dopaminergic neurons in the striatum. We further hypothesize that differential SFC networks indicated above are associated with specific clusters of symptoms observed in PD.

## Materials and Methods

### Subjects and Imaging Data

We extracted rsMRI data of *de novo* PD patients (*n* = 87) from the Parkinson’s Disease Progression Marker Initiative (PPMI)^1^ database as available on 14th January, 2015. For 75 PD patients DAT-SPECT striatal binding ratios were available for the same visits as rsMRI. These data were used for cross-modality correlations described below. A detailed description of the PD cohort used for functional connectivity analyses is provided in **Table 1**. Additionally, we downloaded the first available (screening or baseline) structural MRI (sMRI) and DAT-SPECT scans for all available PD patients [sMRI: *n* = 148 (55 female, mean age ± standard deviation: 61.4 ± 9.4); DAT-SPECT: *n* = 419 (132 female, mean age ± standard deviation: 62.3 ± 9.4)] and age and sex matched healthy controls [sMRI: *n* = 69 (24 female, mean age ± standard deviation: 60.2 ± 11.2); DAT-SPECT: *n* = 198 (63 female, mean age ± standard deviation: 61.4 ± 10.8)]. As resting state was added after the study start some of the PD patients were scanned on PD medication. For some PD patients receiving drug treatment double evaluations of UPDRS part III both in the on and off medication state were available. As all other UPDRS subscales for these patients were acquired in the on state we restricted our analyses to the on medication scores. Written informed consent was obtained from all subjects. The study was approved by Institutional Review Boards/Independent Ethics Committees. For more details on the study please see http://www.ppmi-info.org/wp-content/uploads/2013/02/PPMI-Protocol-AM5-Final-27Nov2012v6-2.pdf.

**Table 1:** Subject group characteristics for resting state SFC analyses.

Group	PD patients	PD patients	PD patients	HC	PD patients	HC
Modality	rsMRI	rsMRI and DAT-SPECT	DAT-SPECT		sMRI	
Analysis	Correlations with UPDRS	Cross-modal correlations	Group comparison		Group comparison	
*N*	87	75	419	198	148	69
Age	61 ± 10.5 [38–78]	60.4 ± 10.6 [38–78]	62.3 ± 9.4	61.4 ± 10.8	61.4 ± 9.4	60.2 ± 11.2
Sex (male/female)	59/28	50/25	287/132	135/63	93/55	45/24
Dominant side (left/symmetric/right)	33/2/52	32/2/41	173/13/233	–	61/3/84	–
UPDRS tot (mean ±*SD* [range])	33.4 ± 15.9 [8–77]	33.4 ± 16.6 [8–77]	32.5 ± 13.6 [7–77]	–	30.1 ± 13.5 [7–70]	–
UPDRS I (mean ± *SD* [range])	6.9 ± 5.1 [0–23]	6.9 ± 5.3 [0–23]	5.9 ± 4.3 [0–22]	–	4.9 ± 3.7 [0–18]	–
UPDRS II (mean ±*SD* [range])	6.8 ± 4.7 [1–23]	6.9 ± 4.9 [1–23]	6.0 ± 4.3 [0–24]	–	5.4 ± 3.9 [0–17]	–
UPDRS III (mean ±*SD* [range])	19.8 ± 10.0 [6–47]	19.6 ± 10.1 [6–47]	20.5 ± 9.0 [5–60]	–	20.5 ± 9.2 [4–42]	–
On L-dopa (yes/no)	32/55	28/47	–	–	–	–
On DA (yes/no)	19/68	15/60	–	–	–	–
On Others (yes/no)	19/68	18/57	–	–	–	–
Resting tremor (yes/no)	66/21	58/17	329/90	–	111/37	–
Rigidity (yes/no/unknown)	77/9/1	66/8/1	316/100/3	–	121/26/1	–
Bradykinesia (yes/no/unknown)	84/2/1	72/2/1	344/72/3	–	133/14/1	–
Postural instability (yes/no/unknown)	3/82/2	2/72/1	31/384/4	–	7/139/2	–

*DA, dopamine agonists; DAT-SPECT, dopamine transporter single photon emission tomography; PD, Parkinson’s disease; rsMRI, resting state magnetic resonance imaging; SD, standard deviation; sMRI, structural magnetic resonance imaging.*

### Image Processing

All image pre-processing steps were performed using the Statistical Parametric Mapping 12 software package (SPM12)^2^ and Matlab R2013.b (MathWorks). In brief, for rsMRI it comprised motion correction, for sMRI data segmentation using NewSegment, spatial normalization to the Montreal Neurological Institute (MNI) space using sMRI based parameters (without modulation for rsMRI to preserve the original signal and with modulation for gray matter probability maps to preserve volumetric information – GMV). Preprocessing of DAT-SPECT data comprised normalization to an average size DAT-SPECT template with subsequent normalization into MNI space. All imaging data were smoothed with Gaussian kernel of 8 mm full-width at half maximum. All analyses were restricted to a gray matter mask derived from the gray matter template provided by SPM (probability of gray matter >0.2).

### Group Comparisons of DAT-SPECT and sMRI Data

The pre-processed DAT-SPECT and GMV images PD patients and healthy controls were entered into voxel-wise general linear models (GLMs) with diagnosis as a factor and controlled for age and sex (**Table 1**). In GMV analyses, total intracranial volume was additionally controlled for. The contrast showing reduced signal in PD was evaluated for both. A voxel-wise family-wise error (FWE) corrected threshold of *p* < 0.05 was applied for these analyses.

### Functional Connectivity of the Striatum

**FIGURE 1:**
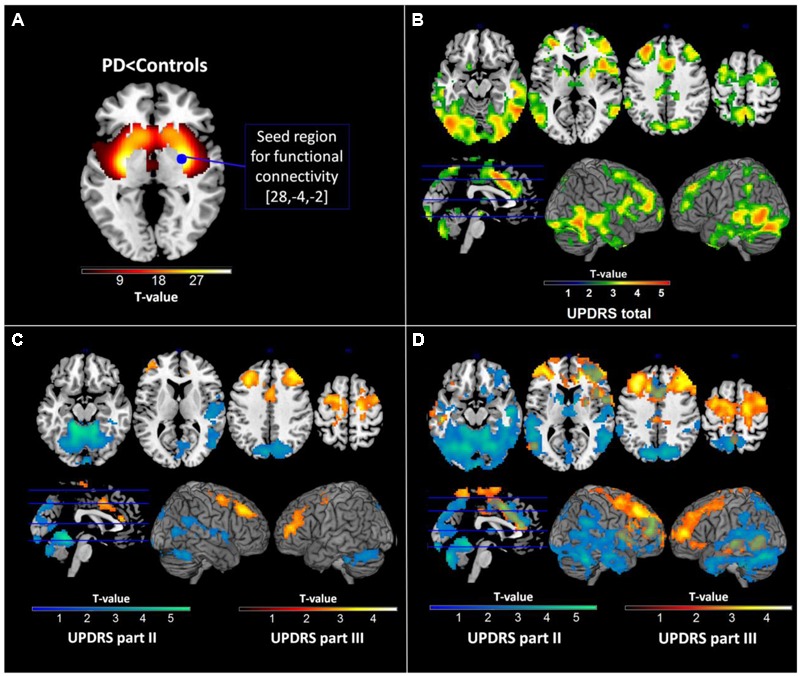
**Results of dopamine transporter single photon emission tomography (DAT-SPECT) and functional connectivity analyses. (A)** DAT-SPECT results showing reduced striatal activity in Parkinson’s disease patients relative to healthy controls and the seed region used for subsequent functional connectivity analyses. **(B)** Regions showing significant negative correlations with the Unified Parkinson’s Disease Rating Scale (UPDRS) total score in the functional connectivity analysis. **(C)** Regions showing significant negative correlations with different parts of the UPDRS in the functional connectivity analysis with all subscales. **(D)** Regions showing significant negative correlations with different parts of the UPDRS in the functional connectivity analysis with separate subscores. All images are displayed in neurological convention.

As motion has been reported to be a critical factor affecting resting state signal (Van Dijk et al., 2012), we regressed out the effects of motion for each subject using a 24 parameter model (6 translational and rotational motion parameters, 6 translational and rotational acceleration parameters, and the squared terms of both) that allows for a rigid control of such data (Yan et al., 2013). As PPMI ensured that only data passing pre-specified quality checks are uploaded to the database no patient had to be excluded due to extensive motion (above 1 voxel-size). Functional connectivity maps were computed using the REST toolbox (Song et al., 2011) implemented in Matlab (MathWorks). A sphere with a radius of 5 mm around the striatal peak voxel providing for DAT-SPECT the maximum difference to healthy controls was used as a seed for computation of Fisher’s z-transformed Pearson correlational maps of the average signal within this region and the rest of the brain (SFC) (**Figure 1A**). We used the default settings in the REST toolbox including removing of a linear trend and application of a low- and high-pass filter restricting the computation to frequencies between 0.01 and 0.08 Hz to reduce contribution of physiological noise.

### Statistical Analysis of rsMRI Data

To facilitate the interpretation of potential correlations between UPDRS subscales and SFC maps, we first aimed to quantify the shared variance across these subscales by computing pair-wise Pearson correlation coefficients. We then aimed to understand how the SFC maps are linked to the UPDRS subscales with and without accounting for the variance shared by the subscales. To address this question, the computed SFC maps were first entered into a voxel-wise GLM including UPDRS parts I, II and III as regressors in the same model therewith accounting for each other’s contribution. To understand the link between UPDRS subscales and SFC maps without controlling for the shared variance, the analysis was then recomputed including each of the subscales and the total score in separate GLMs. All GLMs were tested for negative correlations between SFC and the respective UPDRS regressors. All analyses were further controlled for age, sex, dominant side of symptoms and 3 binary medication status covariates (levodopa, dopamine agonists or other PD medications: all yes/no, no information on dose available in the data base). As we were more interested in the identification of anatomical networks underlying the UPDRS subscales rather than the peak regions associated with each of them, an uncorrected voxel-wise threshold of *p* < 0.01 was applied combined with a whole-brain FWE corrected cluster threshold of *p* < 0.05 adjusted for non-stationarity of smoothness. Recently, Eklund et al. (2016), reported that this type of threshold may lead to an increased false positive rate for fMRI type of analyses. To address this issue we ran 1000 permutations of our parametric design randomly assigning the PD clinical data to individual scans and recomputing the contrasts testing for significant correlations with randomized UPDRS scores at the above voxel- and cluster-wise threshold. This evaluation resulted in 4.9% [99% confidence interval: 4.2–5.8%] of permutations showing significant clusters at the chosen statistical threshold strongly suggesting the validity of the applied multiple comparison correction for our data. We also report the exact *p*-values for the obtained clusters as derived from this null distribution (**Table 2**).

**Table 2:** Regions showing a negative correlation with UPDRS subscales in the multiple regression analysis including all subscales.

Negative correlations	Anatomical region	Cluster size	Exact cluster *p*-value	*T*-value	MNI coordinates
UPDRS II	**Bilateral**: vermis 3–8, cerebellum 3–6 and crus 1 and 2, calcarine sulcus, superior occipital gyrus, fusiform gyrus, cuneus, precuneus**Right**: superior, middle and inferior temporal gyrus, Heschl gyrus, superior parietal lobule	4718	0.007	5.38^∗^	-12 -45 -12
UPDRS III	**Bilateral**: superior and middle frontal gyrus, supplementary motor area, anterior and middle cingulate gyrus, premotor cortex	2119	0.019	3.68	-9 27 33
UPDRS total	**Bilateral**: Vermis 4–8, cerebellum 4–8, cerebellar crus 1 and 2, putamen, pallidum and caudate nucleus, thalamus, posterior hippocampus and parahippocampal cortex, insula, inferior, middle and superior temporal gyrus and temporal pole, primary and supplementary motor cortex, paracentral lobule, primary sensory cortex, cuneus, precuneus, anterior and middle cingulate cortex, superior, middle and inferior frontal gyrus, superior medial frontal gyrus, triangular gyrus, superior parietal gyrus, fusiform gyrus, lingual gyrus, superior and inferior occipital gyrus **Left**: supramarginal gyrus, middle occipital gyrus, inferior parietal gyrus **Right**: operculum	15430	<0.001	5.12^∗^	-42 -66 -21

*MNI, Montreal Neurological Institute space; UPDRS, Unified Parkinson’s Disease Rating Scale. ^∗^Significant at a voxel-wise FWE corrected *p* < 0.05.*

**FIGURE 2:**
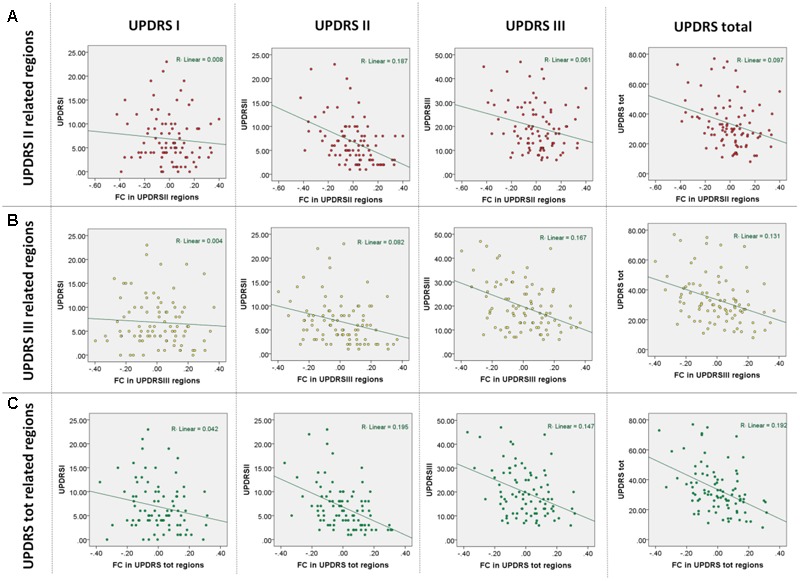
**Results of multiple regression analyses based on functional connectivity regions identified in correlations with the UPDRS subscales. (A)** Observed UPDRS subscales and total scores vs. those predicted in a leave-one-out cross-validation using regions identified as being correlated with UPDRS part II. **(B)** Observed UPDRS subscales and total scores vs. those predicted in a leave-one-out cross-validation using regions identified as being correlated with UPDRS part III. **(C)** Observed UPDRS subscales and total scores vs. those predicted in a leave-one-out cross-validation using regions identified as being correlated with UPDRS total scores.

To quantify the contribution of SFC within the identified networks to the observed symptom severity, we extracted its eigenvariate from all significant clusters adjusted for variance explained by covariates of no interest. To visualize the identified relationships between clinical scales and SFC measures, the eigenvariates from each cluster were plotted against each UPDRS subscale (**Figure 2**).

### Combined Analysis of rsMRI and DAT-SPECT Data

To test if DAT-SPECT and rsMRI indeed provide a differential contribution to clinical symptoms, we performed a mediator analysis. A mediator analysis tests if an initially significant correlation (*p* < 0.05) between two variables is no longer significant after controlling for a third variable that is correlated with both. The third variable is then considered as a mediator between both. In contrast, if the correlation remains significant after controlling for the third variable the correlation between both is considered as independent of the respective variable. For this, partial correlations were computed to test if SFC networks identified in regression analyses with UPDRS subscales and DAT striatal binding explain independent variance in PD symptom severity going beyond the effect of striatal dopaminergic signal loss. The following partial correlations were computed for this purpose (all controlling for age, sex and PD medication status: levodopa, dopamine agonists, other):

(1) Between DAT striatal binding (ipsi- and contralateral putamen and caudate nucleus), UPDRS subscales (UPDRS I, II and III) and SFC cluster eigenvariates identified in voxel-wise regression analyses (SFC clusters showing significant correlations with UPDRS II, III or the total score),
(2) Between UPDRS subscales and SFC eigenvariates controlling for DAT striatal binding,
(3) Between UPDRS subscales and DAT striatal binding controlling for SFC.

Additionally, we formally compared the partial correlation coefficients obtained in (1) between DAT striatal binding and UPDRS subscales and SFC and UPDRS subscales using Steiger’s *z*-test for correlation coefficients (*p* < .05 two-sided) computed on overlapping variables (Wuensch, 2007).

Further, we aimed to better understand the directionality of relationships identified in partial correlation analyses between striatal dopaminergic uptake, SFC and UPDRS. For this, we performed a Bayesian Network analyses using the three phase dependency algorithm implemented in the Matlab-based Causal Explorer toolbox (Cheng et al., 1998; Statnikov et al., 2010). In brief, this algorithm creates a Bayesian network testing for significant causal relationships between any of the variables (*p* < 0.05) in presence of all other included variables. If for example a variable A can be regarded in a causal relationship as a parent or a child of variable B a directed graph is assigned to this relationship. In case no directionality can be derived despite a significant relationship a bidirectional graph is assigned to such connection. The magnitude of the identified associations was quantified using determination coefficients.

## Results

Demographic and clinical description of the rsMRI cohort including the status of PD medication and clinical severity are provided in **Table 1**.

### Results of Group Comparisons for DAT-SPECT and sMRI Data

No atrophic regions were identified in GMV comparisons of PD patients and healthy control subjects. DAT binding in PD was significantly reduced in all striatal regions beside posterior caudate with most pronounced reductions in the right posterior putamen (**Figure 1A**). This region was used as seed for subsequent computations of SFC maps.

### Results of rsMRI Data Analyses

As expected, significant correlations were observed between the UPDRS subscales (UPDRS I and II: *r* = 0.56; *p* < 0.001, UPDRS I and III: *r* = 0.33; *p* < 0.001, UPDRS II and III: *r* = 0.46; *p* < 0.001) indicating that the subscales though sharing up to 31% of variance also provide sufficiently distinct information on disease severity. In the multiple regression analysis of imaging measures with the UPRDS subscales accounting for their shared variance, we found significant negative correlations between SFC and UPDRS part II and III but not with UPDRS part I. Extensive correlations with the UPDRS part II were observed in the vermis and bilateral cerebellar, precuneal, occipital and right temporal and posterior insular regions (this cluster is further referred to as SFC II) (**Table 2**; **Figure 1C**). We also identified significant negative correlations with the UPDRS part III in bilateral premotor, supplementary motor, anterior and middle cingulate, and dorsolateral prefrontal regions (SFC III) (**Table 2**; **Figure 1C**). In correlational analysis with single UPDRS subscales not controlling for the shared variance we found similar but more wide-spread anatomical network for both UPDRS II and III (**Figure 1D**). In regression analysis with UPDRS total scores, significant negative correlations with SFC were detected in regions covering an extensive cortico-subcortical-cerebellar network and including all regions identified for both UPDRS part II and III (SFC total) (**Table 2**; **Figure 1B**). In subsequent correlational analyses, eigenvariates from SFC II, SFC III and SFC total explained up to 19% of variance in these subscales (**Figure 2**).

### Results of Combined rsMRI and DAT-SPECT Data Analyses

**FIGURE 3:**
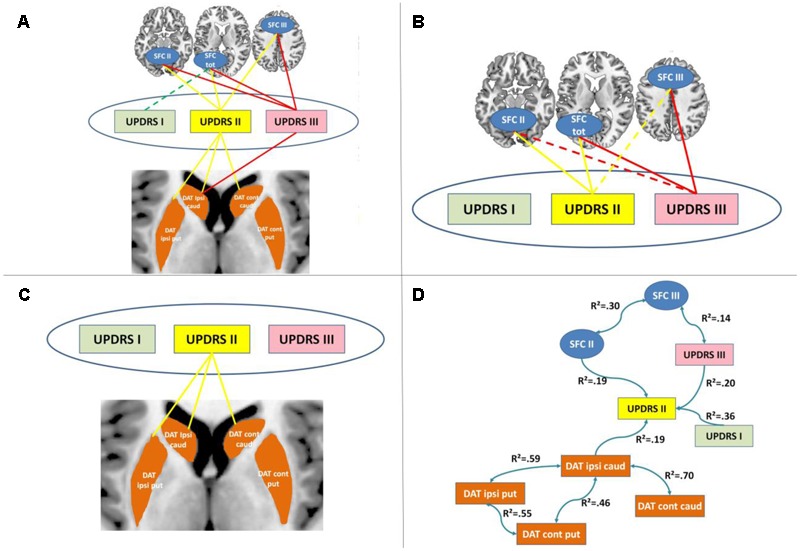
**Results of cross-modal partial correlation and Bayesian network analyses. (A)** Results of partial correlation analyses between imaging and clinical measures when controlling for demographic factors are displayed. Significant partial correlations between the corresponding imaging measures and UPDRS subscales are indicated by solid lines. **(B)** Results of partial correlation analyses between striatal functional connectivity (SFC) and clinical measures when controlling for demographic factors and DAT striatal binding are displayed. Significant partial correlations between the corresponding imaging measures and UPDRS subscales are indicated by solid lines. **(C)** Results of partial correlation analyses between DAT striatal binding and clinical measures when controlling for demographic factors and SFC are displayed. Significant partial correlations between the corresponding imaging measures and UPDRS subscales are indicated by solid lines. **(D)** Results of Bayesian network analyses are displayed. DAT, dopamine transporter; caud, caudate nucleus; put, putamen; SFC I, II and III, striatal functional connectivity regions associated with UPDRS I, II and III, respectively; R^2^, determination coefficient.

In partial correlation analyses only controlling for covariates of no interest but not for shared variance between DAT striatal binding and SFC, the UPRDS I subscale showed a marginally significant correlation with SFC total regions (*r* = -0.22, *p* = 0.071) (**Figure 3A**). As expected, the UPDRS II subscale was significantly correlated with SFC II regions (*r* = -0.46, *p* < 0.001) but also with SFC total (*r* = -0.47, *p* < 0.001), SFC III (*r* = -0.27, *p* = 0.021), with DAT striatal binding in ipsilateral putamen (*r* = -0.31, *p* = 0.009) and in bilateral caudate nucleus (ipsilateral: *r* = -0.36, *p* = 0.002; contralateral: *r* = -0.29, *p* = 0.015). The UPDRS III subscale was significantly correlated with SFC III regions (*r* = -0.42, *p* < 0.001) but also with SFC total (*r* = -0.42, *p* < 0.001), SFC II (*r* = -0.26, *p* = 0.029) and with ipsilateral caudate DAT binding (*r* = -0.25, *p* = 0.034). No significant correlations were observed between SFC and DAT striatal uptake. There was no significant difference between DAT-SPECT and SFC with respect to correlation strength with UPDRS I (*Z* = 0.15, *p* = 0.878), II (*Z* = 0.82, *p* = 0.414) and III (*Z* = 1.17, *p* = 0.244).

In partial correlations between UPDRS subscales and SFC controlling for DAT striatal uptake all significant correlations reported above, except the non-significant correlation between SFC total and UPDRS I (*r* = -0.18, *p* = 0.146), remained significant or marginally significant (UPDRS II and SFC II: *r* = -0.45, *p* < 0.001; UPDRS II and SFC III: *r* = -0.22, *p* = 0.071; UPDRS II and SFC total: *r* = -0.44, *p* < 0.001; UPDRS III and SFC III: *r* = -0.40, *p* = 0.001; UPDRS III and SFC total: *r* = -0.39, *p* = 0.001; UPDRS III and SFC II: *r* = -0.24, *p* = 0.050) (**Figure 3B**).

Similarly, in partial correlations between UPDRS subscales and DAT striatal binding controlling for SFC, all significant correlations between UPDRS II and DAT striatal binding remained significant (UPDRS II and ipsilateral putamen DAT: *r* = -0.27, *p* = 0.028; UPDRS II and ipsilateral caudate DAT: *r* = -0.32, *p* = 0.007; UPDRS II and contralateral caudate DAT: *r* = -0.24, *p* = 0.045) (**Figure 3C**). The initially significant correlation between UPDRS III and ipsilateral caudate DAT binding was no longer significant after controlling for SFC indicating a mediator effect of SFC onto the initially observed association between both (*r* = -0.17, *p* = 0.164).

The Bayesian network analyses revealed an independent contribution of DAT striatal binding and II and III to UDPRS symptoms (**Figure 3D**). More specifically, as expected SFC II was linked to UPDRS II, and SFC III to UPDRS III. DAT striatal binding in ipsilateral caudate nucleus was linked to UPDRS II. All other DAT striatal binding regions were only indirectly linked to UPDRS through this relationship. Interestingly, UPDRS I and III were directionally contributing to UPDRS II scale. No significant link was identified between any DAT striatal binding and SFC regions nor between UPDRS I and any of the imaging measures.

## Discussion

Here we analyzed the association between PD symptom domains, striatal dopaminergic deficits and SFC at rest in the largest currently available cohort of *de novo* PD patients. For the first time, we show that SFC provides an independent and comparably strong link to PD clinical symptoms as the striatal dopaminergic deficits. We also find distinct anatomical SFC networks to contribute to different PD symptom domains as captured by UPDRS part II and III; the first reflecting the ability to perform daily routine automatized motor functions and the second measuring directly clinician-rated motor symptoms associated with PD.

The mediator and Bayesian network analyses confirm the independent contribution of the SFC and dopaminergic deficits to PD clinical symptoms as measured by UPDRS II. More specifically, the Bayesian network analyses suggest that dopaminergic signal and SFC independently contribute to the UPDRS II activities of daily living subscale. In contrast, both the mediator and the Bayesian network analysis suggest a mediator effect of SFC onto the observed relationship between UPDRS III and DAT striatal binding reported in previous studies and reviews (Benamer et al., 2000; Eshuis et al., 2006; Nobili et al., 2010; McGhee et al., 2013). In line with this, the lack of a significant correlation between DAT striatal binding and SFC supports the idea of independent pathological mechanisms underlying both types of measures. Conceptually, DAT binding reflects the loss of dopaminergic projections from substantia nigra into the striatum. However, it does not provide direct information on the resulting downstream perturbations of striato-cortical loops which are presumably more directly reflected by SFC. In example, DAT striatal binding is already substantially reduced in recently diagnosed PD with mild symptom severity (Tissingh et al., 1998). This finding supports the idea of striatal dopaminergic projections not being directly associated with the manifestation of clinical symptoms, i.e., due to compensational mechanisms (Lloyd, 1977).

We find the UPDRS part II being closely related to SFC to vermis, cerebellum and posterior cortical regions, including the temporo-parietal junction. These relationships match well-known brain networks for retention of highly automatized motor functions and bodily self-awareness (Doyon et al., 1998; Lang and Bastian, 2002; Ionta et al., 2011; Serino et al., 2013). In contrast, UPDRS part III is more closely linked to SFC to prefrontal regions associated with motor and executive functions. The lack of significant correlations with UPDRS part I may be related to the higher heterogeneity of functions covered by this subscale and/or involvement of other disease mechanisms (Murai et al., 2001; Goetz et al., 2008). Both identified anatomical networks are strikingly similar to regions reported in previous studies in PD and healthy controls as being functionally connected to the posterior putamen (Helmich et al., 2010; Tziortzi et al., 2014). The prefrontal regions identified in our study are also consistent with regions showing diffusion alterations in PD patients (Kendi et al., 2008). The anatomical networks are also consistent with recent findings reporting these regions to be associated with altered activity and connectivity in PD patients during execution and attention to novel versus automatic movements (Wu et al., 2014). The cerebellar and vermis SFC to the striatum as well as the within-striatum connectivity has been shown to be in general decreased in PD and increased after levodopa administration, supporting the hypothesized involvement of this striato-cerebellar network in generation of some of the PD symptoms (Hacker et al., 2012; Jech et al., 2013; Bell et al., 2015). Also importantly, several studies suggested a potential compensatory role of both cerebellar and premotor synchronization in PD (Wu et al., 2009; Hacker et al., 2012). Our findings are fully in line with these hypothesized mechanisms as one would expect that a reduction or deficit in the compensatory synchronization would lead to increased PD symptoms.

As the same striatal seed region was used for all analyses the identified differences in underlying networks can be therefore regarded as symptom specific desynchronization of posterior putamen activity with corresponding SFC networks. This notion of differential processes involved in generation of PD symptoms is also supported by the relatively low to moderate correlations observed between UPDRS subscales. By controlling our statistical design for different medication and for dominant side of symptoms and testing for potential effects of atrophy we further account for known potential factors affecting functional connectivity in this patient group (Hacker et al., 2012; Jech et al., 2013).

Overall, these findings suggest the potential usability of resting state SFC as a surrogate biomarker endpoint for PD symptoms. However, several further studies will be needed to first validate this identified relationship and to establish longitudinal and potentially mechanistic links between SFC networks and the respective symptoms. Nonetheless, the distinct anatomical networks identified in our study indicate the existence of differential neuronal mechanisms underlying the corresponding PD symptoms. These findings are also in line with results of previous factorial analyses, suggesting contribution of different factors to UPDRS subscales (Stebbins and Goetz, 1998; Goetz et al., 2008). Notably, we find that both anatomical networks associated with UPDRS part II and III also correlate with the UPDRS total score. This finding supports the use of the total score as a composite measure reflecting several PD related pathological processes. However, our results also suggest that caution might be required when using the UPDRS total score for efficacy evaluation of focal treatment procedures such as transcranial magnetic stimulation (Shimamoto et al., 1999; Pal et al., 2010). Based on our results, one would for example expect that a stimulation focus on the premotor region would predominantly result in an improvement of the UPDRS part III but not of the other subscales. This prediction is consistent with the results of a recent large double-blind sham-controlled transcranial magnetic stimulation study reporting an improvement in UPDRS part III but not in UPDRS part I and II after stimulation of the supplementary motor area (Shirota et al., 2013).

We do not identify any statistically significant atrophy in our study in the comparison of *de novo* PD patients and control subjects suggesting a neglectable contribution of atrophy to the observed correlations between functional connectivity and UPDRS subscales (Dukart and Bertolino, 2014). Considering the large sample size included for structural analyses, the lack of findings in our study is unlikely to be a result of insufficient power to detect such effects. The lack of significant atrophy is in contrast to a previous study reporting striatal atrophy in subsample of the cohort included here using a different volumetric estimation algorithm combined with an independent component based approach leading to a more lenient statistical threshold (Zeighami et al., 2015). In a recent meta-analysis of voxel-based morphometry studies in PD the orbitofrontal gray matter volume was identified as consistently reduced in comparisons with healthy controls (Pan et al., 2012). However, all studies included in this meta-analysis targeted more advanced PD populations. Considering the neurodegenerative nature of PD, occurrence of more pronounced atrophy in later stages is therefore likely and might explain the lack of findings in our study. Nonetheless, in line with several previous studies our results strongly question the usability of gray matter atrophy as detected by voxel-based morphometry as an early biomarker of PD (Tessa et al., 2008; Dalaker et al., 2009; Menke et al., 2014).

## Author Contributions

JD designed the study, analyzed the data and wrote the manuscript. FS and AB designed the study and reviewed the manuscript.

## Conflict of Interest Statement

The authors declare that the research was conducted in the absence of any commercial or financial relationships that could be construed as a potential conflict of interest.

## Footnotes

1 http://www.ppmi-info.org/

2 http://www.fil.ion.ucl.ac.uk/spm
